# UHRF1 overexpression promotes osteosarcoma metastasis through altered exosome production and AMPK/SEMA3E suppression

**DOI:** 10.1038/s41389-022-00430-6

**Published:** 2022-09-06

**Authors:** Stephanie C. Wu, Ahhyun Kim, Yijun Gu, Daniel I. Martinez, Loredana Zocchi, Claire C. Chen, Jocelyne Lopez, Kelsey Salcido, Sarah Singh, Jie Wu, Ali Nael, Claudia A. Benavente

**Affiliations:** 1 Department of Pharmaceutical Sciences, University of California, Irvine, CA 92697 USA; 2 Department of Developmental and Cell Biology, University of California, Irvine, CA 92697 USA; 3 Department of Biological Chemistry, University of California, Irvine, CA 92697 USA; 4 Chao Family Comprehensive Cancer Center, University of California, Irvine, CA 92697 USA; 5 Department of Pathology, University of California, Irvine, CA 92697 USA; 6 Department of Pathology, Children’s Hospital of Orange County, Orange, CA 92868 USA

**Keywords:** Bone cancer, Tumour angiogenesis, Metastasis, Oncogenes

## Abstract

Loss-of-function mutations at the retinoblastoma (*RB1*) gene are associated with increased mortality, metastasis, and poor therapeutic outcome in several cancers, including osteosarcoma. However, the mechanism(s) through which *RB1* loss worsens clinical outcome remains understudied. Ubiquitin-like with PHD and Ring Finger domains 1 (UHRF1) has been identified as a critical downstream effector of the RB/E2F signaling pathway that is overexpressed in various cancers. Here, we determined the role and regulatory mechanisms of UHRF1 in rendering osteosarcoma cells more aggressive. Higher UHRF1 expression correlated with malignancy in osteosarcoma cell lines, clinical samples, and genetically engineered mouse models. Gain- and loss-of-function assays revealed that UHRF1 has cell-intrinsic and extrinsic functions promoting cell proliferation, migration, invasion, angiogenesis, and metastasis. UHRF1 overexpression induced angiogenesis by suppressing AMPK activation and Semaphorin 3E (SEMA3E) expression. Further, UHRF1-mediated migration and metastasis resulted, at least in part, through altered expression of extracellular vesicles and their cargo, including urokinase-type plasminogen activator (uPA). Novel osteosarcoma genetically engineered mouse models confirmed that knocking out *Uhrf1* considerably decreased metastasis and reversed the poorer survival associated with *Rb1* loss. This presents a new mechanistic insight into *RB1* loss-associated poor prognosis and novel oncogenic roles of UHRF1 in the regulation of angiogenesis and exosome secretion, both critical for osteosarcoma metastasis. This provides substantial support for targeting UHRF1 or its downstream effectors as novel therapeutic options to improve current treatment for osteosarcoma.

## Introduction

Osteosarcoma is the most common primary bone cancer in children and young adults. As a highly metastatic cancer, ~20% of osteosarcoma patients are diagnosed after cancer has already metastasized (typically to the lungs), which translates to 5-year survival rates of <40% [1]. Unfortunately, pulmonary metastases occur in nearly half of osteosarcoma patients [2]. Thus, there is a pressing clinical need to determine the molecular mechanisms responsible for metastasis in osteosarcoma to facilitate the development of new therapeutic strategies.

The recurrent genetic mutations found in osteosarcoma are in tumor suppressors *TP53* and *RB1* [3, 4]. Loss-of-function mutations in *RB1* are associated with poor therapeutic outcome: increased mortality, metastasis, and poor response to chemotherapy [5–9]. However, the mechanism(s) through which *RB1* loss leads to poor prognosis remain unclear. We previously identified Ubiquitin-like, containing PHD and RING finger domains 1 (UHRF1) as a target of interest downstream of the RB/E2F signaling pathway [10]. UHRF1 is a multidomain protein that exerts various functions that include reading and writing of epigenetic codes. UHRF1 is most known for its role in maintaining DNA methylation [11, 12]. UHRF1 is overexpressed in different cancers that frequently present *RB1* mutations, including lung, breast, bladder, colorectal cancer, and retinoblastoma [10, 13–16]. This suggests that UHRF1 upregulation may contribute to tumor progression following *RB1* loss in osteosarcoma.

Here, we observed that UHRF1 is critical in osteosarcoma tumorigenesis, promoting proliferation, migration, angiogenesis, and metastasis. Mechanistically, we show that high UHRF1 decreases Semaphorin 3E (SEMA3E) expression through the suppression of AMPK activation, resulting in increased induction of angiogenesis. We also identified a novel role of UHRF1 in promoting exosome secretion which is associated, at least in part, with migration and invasion through the secretion of proteins, including urokinase-type plasminogen activator (uPA). Targeting uPA with small-molecule inhibitors resulted in a robust decrease in osteosarcoma migration and invasion. *Uhrf1* loss drastically delayed tumor onset, decreased pulmonary metastasis, and increased the lifespan of developmental osteosarcoma mouse models carrying *Rb1* mutation. These findings highlight a critical role of UHRF1 as a driver of the poor prognosis associated with *RB1* loss and present UHRF1, and its downstream targets, as novel osteosarcoma therapeutic targets.

## Results

### UHRF1 expression correlates with osteosarcoma malignancy and metastasis

**Fig. 1:**
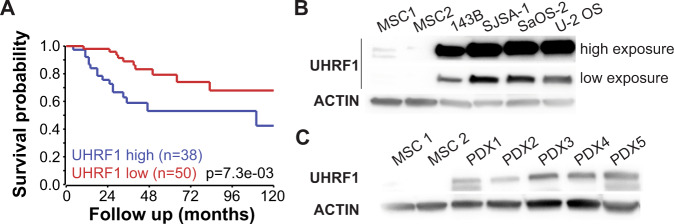
UHRF1 expression directly correlates with malignancy and metastasis in osteosarcoma. **A** Overall survival probability of osteosarcoma patients comparing tumors with high *UHRF1* (blue, *n* = 38) versus low *UHRF1* (red, *n* = 50) expression at the time of diagnosis. **B**, **C** Western blot analysis of UHRF1 in (**B**) human osteosarcoma cell lines compared to MSC as control and (**C**) PDXs tumors. High exposure was used for UHRF1 detection in MSCs. β-actin was used as a loading control.

The Cancer Genome Atlas (TCGA) data suggest *UHRF1* is significantly overexpressed in malignancies that frequently present RB pathway inactivation including sarcomas, breast invasive carcinomas, and lung adenocarcinomas, but not in cancers where *RB1* loss is infrequent, like prostate adenocarcinomas (Supplementary Fig. 1A) [17]. In sarcomas, high *UHRF1* expression is associated with poorer survival (Supplementary Fig. 1B). *UHRF1* expression analysis using published RNA-Seq datasets from pretreatment biopsies from 88 osteosarcoma patients [18] associate high *UHRF1* expression with poorer overall survival (Fig. 1A). *UHRF1* mRNA in situ hybridization on an osteosarcoma tissue array confirmed a correlation between stage of malignancy and *UHRF1* expression (Supplementary Fig. 1C, D).

The clinical data led to examine UHRF1 mRNA and protein levels in 4 human osteosarcoma cell lines: 143B, SJSA-1, SaOS-2 (*RB1-*null cell line), and U-2 OS, and 5 patient-derived orthotopic xenografts (PDX1-5). qPCR analysis showed significant upregulation of *UHRF1* mRNA levels in all samples except PDX2 when compared to MSC, independent of RB1 expression (Supplementary Fig. 1E–H). At the protein level, UHRF1 was highly expressed across all samples compared to MSC (Fig. 1B, C).

### *UHRF1* is a direct target of the RB/E2F signaling pathway in the osteogenic lineage

UHRF1 is reported as a direct E2F1 transcriptional target [19, 20]. Chromatin immunoprecipitation (ChIP) analysis using mesenchymal stem cells (MSCs) confirmed E2F1 enrichment at consensus binding motifs within the *UHRF1* promoter (Supplementary Fig. 2A). However, knocking down E2F1 alone was insufficient to decrease UHRF1 expression in osteosarcoma cells (Supplementary Fig. 2B). We tested compensation by other activator E2Fs and found that knocking down E2F1 alongside E2F3, but not E2F2, reduced UHRF1 expression in osteosarcoma (Supplementary Fig. 2C, D). E2F3 knockdown alone was also insufficient to decrease UHRF1 expression (Supplementary Fig. 2E). Treatment with palbociclib, a CDK4/6 inhibitor, decreased *UHRF1* mRNA and protein levels in all cell lines except SaOS-2, which is *RB1*-null and served as negative control (Supplementary Fig. 2F, G). Increased UHRF1 expression in *RB1*-wild-type cells correlated with upregulation of *CDK4* and/or *CDK6* transcripts and/or *INK4A* downregulation (Supplementary Fig. 2H, I). *CDK4* amplification occurs in SJSA-1 and *INK4A* deletion in U2-OS [21, 22]. Thus, the RB/E2F pathway regulates *UHRF1* through direct transcriptional activation by activator E2Fs, E2F1, and E2F3.

### UHRF1 overexpression promotes osteosarcoma cell proliferation in vitro and in vivo

**Fig. 2:**
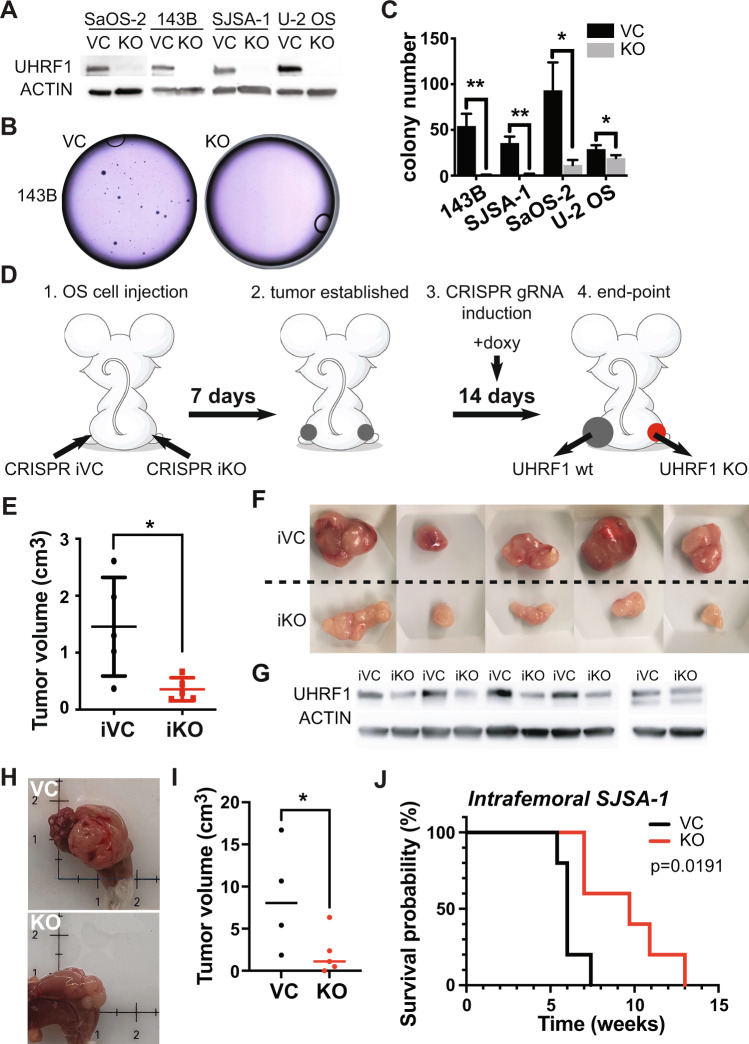
UHRF1 promotes osteosarcoma tumor growth in vitro and in vivo. **A** Western blot analysis of UHRF1 in CRISPR/Cas9-mediated UHRF1 knockout of osteosarcoma cell lines (KO) in comparison to non-targeting vector control (VC). β-actin was used as a loading control. **B** Representative images from clonogenic assay plates with 143B VC and UHRF1 KO. **C** Histogram of colony counts from clonogenic assay. *P < 0.05, **P < 0.01 by unpaired two-tailed *t* test. **D** Cartoon representation for doxycycline-inducible in vivo knockout for flank-injected osteosarcoma cell lines carrying a non-targeting sgRNA (iCRISPR VC) or UHRF1 sgRNA (iCRISPR KO). **E** Quantification of the tumor volume for each of the replicates. *P < 0.05 by paired two-tailed *t* test. **F** Images from tumors collected from subcutaneous injection of iCRISPR VC (iVC, *n* = 5) and iCRISPR KO (iKO, *n* = 5) SJSA-1. **G** Western blot verification of UHRF1 knockout in iKO compared to iVC for tumors shown in (**F**). β-actin was used as a loading control. **H** Representative images from tumors collected from intrafemoral injection of SJSA-1 VC and UHRF1 KO. **I** Quantification of the tumor volume for each of the replicates, 5 weeks after intrafemoral injection. A solid linerepresents median from *n* = 4 for VC and *n* = 5 for KO. *P < 0.05 by paired two-tailed *t* test. **J** Kaplan–Meier curves showing the survival of mice with orthotopic SJSA-1 VC (black, *n* = 5) and UHRF1 KO (red, *n* = 5) xenografts.

Emerging reports link UHRF1 overexpression in cancer with proliferation, migration/invasion, or both [10, 23–29]. To study the role of UHRF1 in osteosarcoma, we generated syngeneic *UHRF1* CRISPR knockout clones (KO) and non-targeting vector controls (VC). Successful UHRF1 KO was achieved in all four osteosarcoma cell lines tested (Fig. 2A). Proliferation and EdU-incorporation analyses indicated UHRF1 KO cells have longer doubling times and lower proliferation rates compared to VC (Supplementary Fig. 3). This decrease in proliferation was confirmed by clonogenic assays showing significant reductions in both the number and the size of colonies in UHRF1 KO compared to VC cells (Fig. 2B, C).

To assess proliferative capacity in vivo, SJSA-1 UHRF1 KO and VC cells were injected subcutaneously at opposite flank regions. UHRF1 KO cells gave rise to significantly smaller tumors (average size 0.49 ± 0.37 cm^3^) when compared to VC (average volume 1.23 ± 0.75 cm^3^; *P* = 0.02; *n* = 10). The therapeutic potential of targeting UHRF1 in established tumors was tested using a doxycycline-inducible system to drive the expression of CRISPR/Cas9 gRNA against UHRF1. Inducible UHRF1 KO (iKO) and inducible VC (iVC) osteosarcoma cells were subcutaneously injected, and tumors allowed to establish for one week before inducing Cas9 expression via oral doxycycline delivery (Fig. 2D). Tumor growth was significantly reduced in UHRF1 iKO tumors (average tumor volume 0.356 ± 0.201 cm^3^; *n* = 5) compared to iVC tumors (1.455 ± 0.865 cm^3^; *P* = 0.02; *n* = 5) (Fig. 2E, F). Western blot analyses confirmed reduced UHRF1 protein at varying levels in UHRF1 iKO tumors (Fig. 2G). Thus, it is possible that residual tumors may be formed from cells not exposed to doxycycline or with incomplete UHRF1 KO. UHRF1 iKO tumors presented phenotypic features of less aggressive tumors, with lessened vasculature and ulceration compared to iVC tumors (Fig. 2F). Induction of a different UHRF1 gRNA in SJSA-1 resulted in similar tumor size reduction, as well as with other osteosarcoma cell lines (Supplementary Fig. 4A–D).

An orthotopic model using intrafemorally injected SJSA-1 confirmed UHRF1 KO tumors grow slower and have significantly higher survival than VC (Fig. 2H–J and Supplementary Fig. 4E). Together, our data suggest UHRF1 is critical for osteosarcoma growth.

### UHRF1 promotes osteosarcoma metastasis

**Fig. 3:**
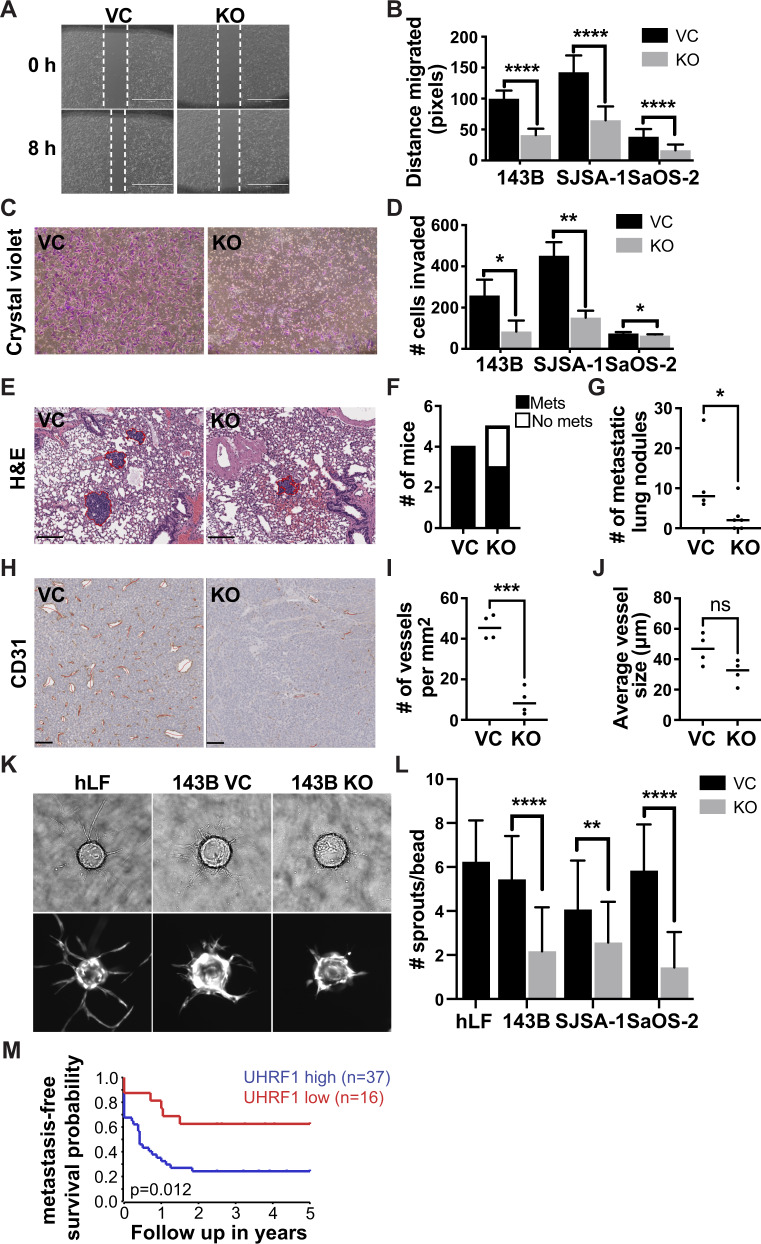
UHRF1 promotes osteosarcoma cell migration and invasion in vitro and in vivo. **A** Representative image from scratch-wound assay comparing wound closure of non-targeting vector control (VC) and UHRF1 KO (KO) cells over 8 h in SJSA-1 cells. White dashed lines represent the wound edge. Scale bar, 1000 μm. **B** Quantification of distance (pixels) migrated in scratch-wound assays for each of the osteosarcoma cell lines. Each data point is mean ± s.d. of ten measurements in triplicate samples. **C** Representative images from Transwell assays with cells stained with crystal violet (purple) comparing levels of invasion between VC and UHRF1 KO in SJSA-1 cells. **D** Quantification of the number of cells invaded across the Transwell membrane for each of the osteosarcoma cell lines. Each data point is mean ± s.d. of triplicate samples. **E** Representative histological images of H&E-stained lung section from intrafemoral-injected mice with VC and UHRF1 KO SJSA-1 cells, 5 weeks after injection. Scale bar, 200 μm. Quantification of the burden of lung metastases was quantified as (**F**) the number of mice with or without lung metastases and (**G**) the number of metastatic lung nodules per mouse. A solid line represents median from *n* = 4 for VC and *n* = 5 for KO. *P = 0.03 by unpaired *t* test. **H** Representative histological images of CD31 expression in intrafemoral tumors derived from VC and UHRF1 KO SJSA-1 cells, 5 weeks after injection and (**I**) quantification of the average number of vessels per mm^2^ and (**J**) quantification of the mean vessel diameter. Solid line represents median from *n* = 4. ns not significant; ***P = 0.002 by unpaired *t* test. Scale bars, 100 μm. **K** Representative brightfield (top) and fluorescent (bottom) images of late phase sprouting angiogenesis assay (day 6) using human lung fibroblasts as assay control and 143B VC and KO conditioned media in the fibrin gel bead assay. This is quantified as (**L**) the number of sprouts per bead. *n* = 30. **M** 5-year metastasis-free survival probability of osteosarcoma patients comparing tumors with high *UHRF1* (blue, *n* = 37) versus low *UHRF1* (red, *n* = 16) expression at the time of diagnosis. For all graphs: *P < 0.05, **P < 0.01, ****P < 0.0001 by unpaired two-tailed *t* test.

The high metastatic potential of osteosarcoma is the main cause of mortality in patients. UHRF1 was reported to promote cell invasion following exogenous UHRF1 overexpression in osteosarcoma [29]. Here, we evaluated whether the endogenously high UHRF1 protein levels present in osteosarcoma promote cell migration, invasion, angiogenesis, and metastasis. Scratch-wound assays were used to assess migration (Fig. 3A). UHRF1 KO resulted in a significant decrease in cell migration, with an average 56.8 ± 2.3% reduction in migration across three cell lines (Fig. 3B). Invasion assays also revealed a significant reduction in the number of UHRF1 KO cells capable of mobilizing across Matrigel-coated inserts (Fig. 3C, D).

To determine if UHRF1 overexpression is sufficient to induce cell migration, we performed gain-of-function studies in MSCs. Using a doxycycline-inducible system to drive UHRF1 overexpression (pCW57-UHRF1; Supplementary Fig. 6A, B), we observed a significant increase in MSC migration and invasion in cells induced to overexpress UHRF1 compared to controls (Supplementary Fig. 5C–F). We also utilized our pCW57-UHRF1 vector to rescue UHRF1 expression in UHRF1 KO cells. Doxycycline treatment for 24 h was able to restore UHRF1 protein expression back to levels comparable to wild-type and significantly increase migration in all osteosarcoma cell lines (Supplementary Fig. 5G, H).

The reduction in migration and invasion observed in vitro translated into a lessened metastatic potential in vivo. We assessed the rate of spontaneous lung metastasis in the NSG mice bearing intrafemoral SJSA-1 UHRF1 KO and VC tumors (Fig. 2H, I). At 5 weeks after injection, 100% of VC mice (4/4 mice) had metastatic lung nodules compared to only 60% of UHRF1 KO mice (3/5 mice) and the number of metastatic lung nodules was significantly lower in UHRF1 KO compared to VC (Fig. 3E–G). To test if UHRF1 contributes to tumor intravasation or extravasation, SJSA-1 UHRF1 KO and VC cells were injected into the tail vein of NSG mice to assess the rate of lung colonization. At 3-weeks post injection, UHRF1 KO mice had fewer lung metastatic nodules, but only the nodule size was significantly smaller than VC (Supplementary Fig. 6A–F).

Following the observation that UHRF1 loss appears to reduce tumor vascularization (Fig. 2F, H), we probed UHRF1 VC and KO orthotopic xenografts for the vasculature marker CD31. UHRF1 KO tumors exhibited reduced number of tumor vessels without reducing the overall tumor vessel caliber compared to VC (Fig. 3H–J). Thus, we evaluated a novel role of UHRF1 in cancer cell induction of endothelial cell migration (sprouting) using a fibrin gel bead assay (Fig. 3K). UHRF1 KO osteosarcoma cells displayed a significant lower ability to induce endothelial cell sprouting compared to VC (Fig. 3L). These data suggest a novel role of UHRF1 in osteosarcoma cell’s ability to affect early stages of angiogenesis.

*UHRF1* expression analysis using pretreatment biopsies from 53 osteosarcoma patients [18] associate low *UHRF1* expression with increased rates of 5-year metastasis-free survival (Fig. 3M). Together, these results support the role of UHRF1 in the early stages of osteosarcoma metastasis, including tumor migration, invasion, and angiogenesis, and promotes tumor growth at secondary sites.

### High *PLAU*/uPA expression is associated with enhanced osteosarcoma cell migration and invasion

Given UHRF1 role in heterochromatin maintenance, we analyzed the effect of UHRF1 loss on DNA methylation, chromatin structure, and transcription to determine how UHRF1 serves as an oncogene in osteosarcoma. Decreased genomic DNA methylation was detected in UHRF1 KO compared to VC in most osteosarcoma cells examined (Supplementary Fig. 6A). Reduced representation bisulfite sequencing (RRBS) analysis of differentially methylated regions across the genome of VC and UHRF1 KO cells confirmed a reduction in DNA methylation, with most changes occurring at non-coding regions of the genome (Supplementary Fig. 8B). We also assessed chromatin landscape changes upon UHRF1 loss by performing ATAC-seq on UHRF1 KO compared to VC (Supplementary Fig. 8C). Despite the role of UHRF1 in heterochromatin maintenance and decreased genomic DNA methylation levels, we identified only 16 chromatin regions with significant changes in chromatin accessibility across three biological replicates (Supplementary Table 1). Within these changes, the majority were consistent with the role of UHRF1 in chromatin repression, with ~69% (11/16) resulting in the opening of chromatin upon UHRF1 loss.

**Fig. 4:**
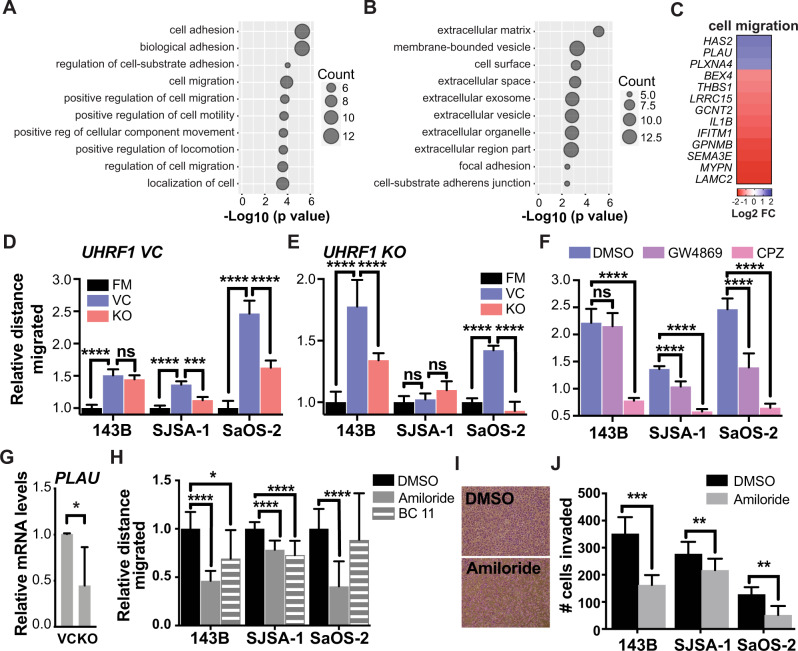
UHRF1 controls the expression of genes involved in exosomes and urokinase plasminogen activator production to drive osteosarcoma migration. **A**, **B** Gene ontology (GO) analysis for (**A**) biological processes and (**B**) cellular components of the downregulated differentially expressed genes (DEGs) identified through RNA-seq. **C** Heatmap of top 13 genes involved in the regulation of cell migration, decreased (blue) or increased (red) in expression level upon UHRF1 loss. **D**, **F** Quantification of relative distance migrated in scratch-wound assays for (**D**) osteosarcoma VC cells assayed with fresh media (FM) or conditioned media (24 h) from VC cells or UHRF1 KO cells, normalized to FM; **E** osteosarcoma UHRF1 KO cells assayed with FM or conditioned media from VC cells or UHRF1 KO cells, normalized to FM; **F** each of the osteosarcoma cell lines treated with DMSO, 10 µM GW4869 or 20 µM CPZ, normalized to DMSO control. Each data point is mean ± s.d. of ten measurements in triplicate samples. **G** qPCR analysis of *PLAU* mRNA levels in flank-implanted tumors from control (VC) and UHRF1 KO (KO) SJSA-1 cell lines. *P < 0.05 by paired two-tailed *t* test. **H** Quantification of relative distance migrated in scratch-wound assays for each of the osteosarcoma cell lines treated with DMSO, 150 µM amiloride, or 16.4 µM BC11 hydrobromide, normalized to DMSO control. Each data point is mean ± s.d. of ten measurements in triplicate samples. **I** Representative images from Transwell assays with cells stained with crystal violet comparing levels of invasion between cells treated with DMSO or 150 µM amiloride in SJSA-1. **J** Quantification of the number of cells invaded in Transwell invasion assay for each of the osteosarcoma cell lines treated with DMSO or 150 µM amiloride. Each data point is mean ± s.d. of triplicate samples. For all graphs: ns not significant, **P < 0.01, ***P < 0.001, ****P < 0.0001 by unpaired two-tailed *t* test.

In line with modest changes in DNA methylation and chromatin accessibility, transcriptome analysis using RNA-seq revealed that the gene expression profile from UHRF1 KO cell lines present minor variability from VC cells (Supplementary Fig. 8D). We identified 272 differentially expressed genes (DEGs; 191 upregulated and 81 downregulated) in UHRF1 KO compared to VC cells. Gene ontology (GO) analysis for biological processes of the DEGs revealed an enrichment of genes involved in the regulation of cell adhesion and migration (Fig. 4A). GO analysis of cellular components of the DEGs from the RNA-seq data also exposed an enrichment of genes involved in extracellular vesicle formation and cell adhesion (Fig. 4B). We confirmed the changes in gene expression observed among top the 13 genes involved in the regulation of cell migration (Fig. 4C) through qPCR (Supplementary Fig. 6E).

The potential role of UHRF1 in migration and invasion through the control of exosome-mediated pathways was examined by testing the effect of conditioned media collected from VC and UHRF1 KO cell cultures on osteosarcoma cell migration. Conditioned media from osteosarcoma VC cells significantly increased autologous osteosarcoma cell migration compared to fresh media (Fig. 4D, blue bars). While conditioned media derived from osteosarcoma UHRF1 KO cells also increased cell migration compared to fresh media, VC cell migration was lower when exposed to UHRF1 KO conditioned media than autologous VC conditioned media (Fig. 4D, red bars). Interestingly, UHRF1 KO cells also displayed a robust increase in cell migration when exposed to VC conditioned media, but not when exposed to autologous UHRF1 KO conditioned media (Fig. 4E). Thus, UHRF1 loss decreases the secretion of pro-migratory factors to the extracellular matrix that can serve as an autologous signal to stimulate osteosarcoma cell migration but does not affect the ability of the cells to respond to these extracellular factors. Inhibition of exosome biogenesis/release using GW4869 and inhibition of endocytosis using chlorpromazine (CPZ) significantly reduced the cell migration induced by osteosarcoma conditioned media (Fig. 4F). Together, this indicates that UHRF1 overexpression alters the production and/or secretion of exosomes and/or its cargo which contribute, at least in part, to increased osteosarcoma cell migration.

Plasminogen activator, urokinase (*PLAU*) was one of the DEGs identified through transcriptome analysis as potentially involved in UHRF1-mediated cell migration. Encoded by *PLAU*, uPA is associated with cell migration and is a known cargo protein in osteosarcoma-secreted exosomes [30]. *PLAU* transcript levels were significantly decreased in UHRF1 KO osteosarcoma cells (Supplementary Fig. 6E) and subcutaneous xenografts, with an average 2.2-fold decrease compared to VC cell-derived tumors (*P* = 0.038, Fig. 4G). Thus, we tested whether uPA inhibition could decrease migratory potential and invasiveness of osteosarcoma cells. Amiloride treatment resulted in significantly reduced migration, with an average 40.3 ± 11.9% decrease across all osteosarcoma cells examined (Fig. 4H). BC11 hydrobromide treatment, a selective uPA inhibitor, resulted in a 23.2 ± 10.2% decrease in migration across all lines (Fig. 4H). Reduced invasiveness was also observed upon uPA inhibition (Fig. 4I, J). Further, amiloride treatment also inhibited cell migration in MSCs with induced UHRF1 expression (Supplementary Fig. 7). Thus, uPA is an important contributor of UHRF1-induced migration in osteosarcoma.

### UHRF1 repression of AMPK activation and SEMA3E expression induces angiogenesis

**Fig. 5:**
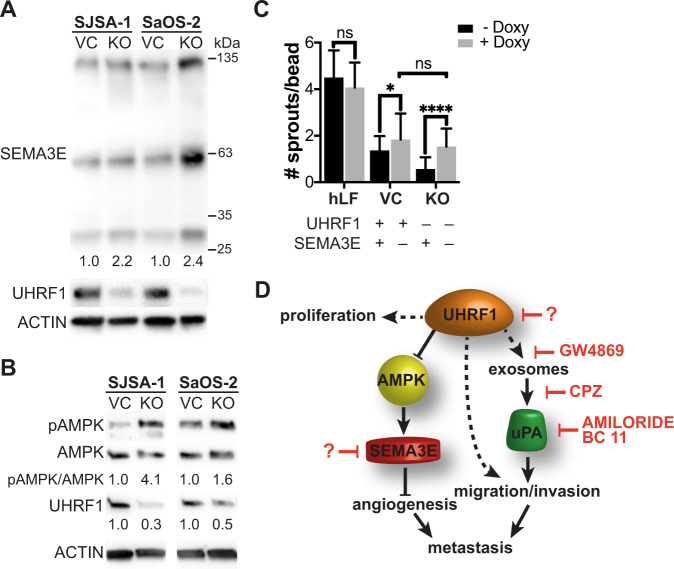
UHRF1 decreases SEMA3E expression through suppression of AMPK activation to induce angiogenesis. **A**, **B** Western blot analysis of (**A**) SEMA3E in SJSA-1 and SaOS-2 VC and KO osteosarcoma cell lines quantification of KO normalized to VC; and (**B**) activated AMPK (pAMPK) and total AMPK with the quantification of the ratio. UHRF1 used to confirm VC and KO status. β-actin was used as a loading control. **C** Early phase sprouting angiogenesis (day 3) using human lung fibroblasts as assay control and SJSA-1 VC and KO cells with inducible sgRNA to knockout SEMA3E upon doxycycline addition in the fibrin gel bead assay. Quantified as the number of sprouts per bead. *n* = 30. ns not significant, *P < 0.05, ****P < 0.0001, by unpaired *t* test. **D** Model of UHRF1 oncogenic function in osteosarcoma. UHRF1 overexpression stimulates proliferation, exosome and uPA production that stimulates migration, invasion, and metastasis. UHRF1 also suppresses AMPK activation to inhibit SEMA3E and induce angiogenesis. Downstream of UHRF1, inhibitors of exosome secretion (e.g., GW4869), exosome endocytosis (CPZ), or uPA inhibitors (e.g., amiloride, BC11 hydrobromide) are attractive therapeutic options to decrease migration and metastasis. The development of UHRF1-targeted therapeutics might result in a beneficial decrease in both tumor growth and pulmonary metastases.

Another DEG identified in our RNA-seq analysis was *SEMA3E* (Fig. 4C). Sema3E acts as a repulsive factor for plexin-D1-expressing endothelial cells, leading to decreased neoangiogenesis and reduced tumor growth [31]. We confirmed that the increased *SEMA3E* transcript expression correlated with increased SEMA3E protein levels in the KO cells compared to VC (Fig. 5A). A recent study identified that UHRF1 suppresses AMPK activity [32], and AMPK activation was shown to induce *SEMA3E* expression [33]. Thus, we tested whether increased SEMA3E expression is correlated with increased phosphorylated AMPK (pAMPK) levels in UHRF1 KO cells. We found that while the total AMPK is unaltered by UHRF1 loss, pAMPK is elevated in UHRF1 KO cells, confirming a direct correlation between AMPK activity and SEMA3E expression (Fig. 5B). To determine if elevated SEMA3E is, at least in part, associated with the lower ability of UHRF1 KO cells to induce angiogenesis (Fig. 3N), we next tested whether knocking out *SEMA3E* could reverse the decreased angiogenesis observed in UHRF1 KO cells. Inducing *SEMA3E* KO resulted in a significant increase in the number of endothelial cells sprouting on the surface of fibrin gel beads both in UHRF1 VC and KO cells (Fig. 5C). Interestingly, upon *SEMA3E* KO, the extent of angiogenesis induced by UHRF1 VC and KO cells is similar (Fig. 5C). This suggests decreased SEMA3E secretion is associated with increased angiogenesis upon UHRF1 overexpression.

### UHRF1 overexpression is a critical driver of metastasis and the poor survival observed in *Rb1*-null osteosarcoma

**Fig. 6:**
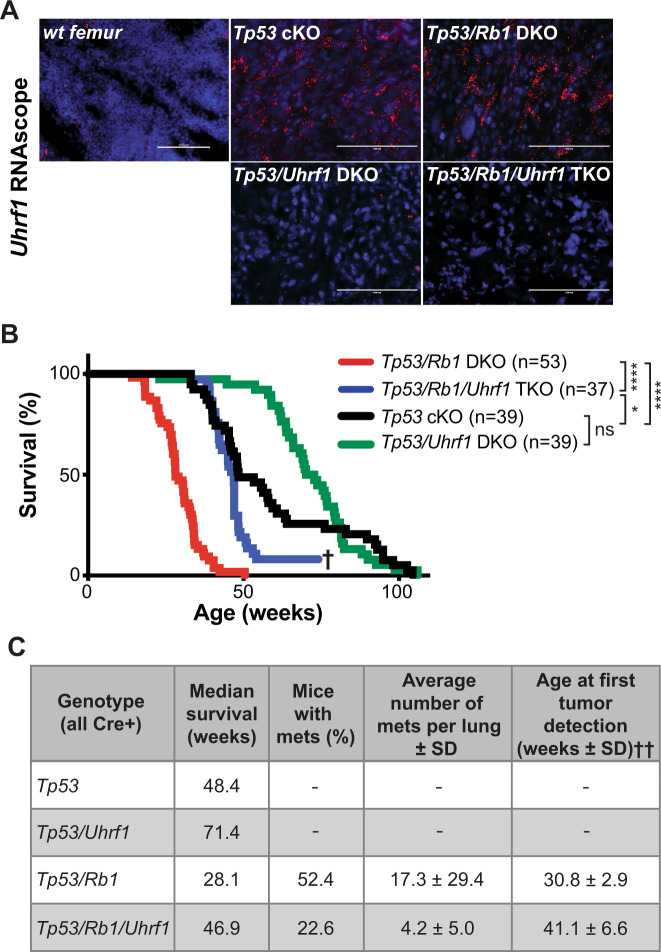
UHRF1 is a critical driver of the increased malignancy observed in RB-null osteosarcoma. **A** Representative fluorescent images of in situ hybridizations using RNAscope on wild-type femurs or tumors from genetically engineered osteosarcoma mice using a probe against *Uhrf1* (red). Nuclei were counterstained with DAPI (blue). *Uhrf1* expression is detected in *Tp53* cKO and *Tp53/Rb1* DKO mouse tumors but is low or not detected in *wt, Tp53/Uhrf1* DKO, and *Tp53/Rb1/Uhrf1* TKO tumors. **B** Kaplan–Meier curves showing the survival of osteosarcoma mouse models. Mice bearing *Rb1* mutations *Tp53/Rb1* DKO: *Osx-Cre; p53*^*lox/lox*^*; Rb1*^*lox/lox*^ (red; *n* = 53) have significantly shorter lifespan compared to *Tp53* cKO: *Osx-Cre; p53*^*lox/lox*^*; Rb1*^*lox/lox*^ (black; *n* = 39). This survival time was significantly increased in *Tp53*/*Rb1*/*Uhrf1* TKO: *Osx-Cre; p53*^*lox/lox*^*; Rb1*^*lox/lox*^; *Uhrf1*^*lox/lox*^ (blue; *n* = 37) mice. ^†^Three surviving mice were removed from the study to confirm tumor absence. *Tp53*/*Uhrf1* DKO: *Osx-Cre; p53*^*lox/lox*^*; Uhrf1*^*lox/lox*^ (green; *n* = 39) showed an overall survival comparable to *Tp53* cKO (black; *n* = 39). Mantel–Cox test were used for curve comparisons. ns not significant, *P < 0.05, ****P < 0.0001. **C** Summary table for each osteosarcoma mouse model. ^††^Age of mice (weeks) at earliest tumor detection via microCT and PET scans comparison: *P* < 0.05 by unpaired two-tailed *t* test; *Tp53/Rb1* DKO (*n* = 4) and *Tp53*/*Rb1*/*Uhrf1* TKO (*n* = 3).

Finally, we evaluated UHRF1’s pro-oncogenic function in osteosarcoma tumorigenesis using developmental mouse models. Loss of *Tp53* in conditional knockout mice driven by osterix-cre recombinase (*Tp53* cKO; *Osx-cre Tp53*^lox/lox^), a transgene expressed in preosteoblasts, results in osteosarcoma formation with complete penetrance [34, 35]. This model is potentiated by *Rb1* loss (*Tp53*/*Rb1* DKO; *Osx-cre Tp53*^lox/lox^ *Rb1*^lox/lox^), mimicking the poor clinical outcome of *RB1* loss in human osteosarcoma [34, 35]. Analysis of osteosarcoma tumors arising from *Tp53* cKO and *Tp53*/*Rb1* DKO mice revealed that UHRF1 is highly expressed at the mRNA (Fig. 6A) and protein level (Supplementary Fig. 8B) in these mouse models.

*Uhrf1* KO mice result in embryonic lethality [36]; thus, we generated *Uhrf1* cKO mice (*Osx-cre Uhrf1*^lox/lox^), *Tp53*/*Uhrf1* DKO (*Osx-cre Tp53*^lox/lox^ *Uhrf1*^lox/lox^) and *Tp53*/*Rb1*/*Uhrf1* triple knockout (*Tp53*/*Rb1*/*Uhrf1* TKO; *Osx-cre Tp53*^lox/lox^ *Rb1*^lox/lox^ *Uhrf1*^lox/lox^) to study the role of UHRF1 in osteosarcoma development. *Uhrf1* cKO mice display normal bone development (Supplementary Fig. 8C, D). For *Tp53*/*Uhrf1* DKO and *Tp53*/*Rb1*/Uhrf1 TKO mice, we tracked overall survival and tumor formation compared to the corresponding littermate controls (*Tp53* cKO and *Tp53/Rb1* DKO, respectively). In situ hybridization confirmed reduced *Uhrf1* expression in *Tp53*/*Uhrf1* DKO and *Tp53*/*Rb1*/*Uhrf1* TKO osteosarcoma tumors (Fig. 6A). All strains presented osteosarcoma with 100% penetrance but with distinct survival rates and disease presentation (Fig. 6B, C). Consistent with previous reports, *Rb1* loss in *Tp53*/*Rb1* DKO mice resulted in shorter median survival compared to *Tp53* cKO mice (Fig. 6B). Strikingly, *Uhrf1* genetic ablation in *Tp53/Rb1/Uhrf1* TKO mice resulted in significantly increased survival compared to *Tp53/Rb1* DKO mice. Further, the median survival of *Tp53/Rb1/Uhrf1* TKO was comparable to *Tp53* cKO (*P* = 0.0577, Gehan–Breslow–Wilcoxon test), suggesting that *Uhrf1* overexpression is critical for the poor prognosis associated with *Rb1* loss (Fig. 1B). However, the overall survival of *Tp53/Rb1/Uhrf1* TKO and *Tp53* cKO mice is distinct (*P* = 0.0126, Mantel–Cox test), suggesting that early UHRF1 overexpression is not the only factor contributing to *Rb1* loss-associated outcomes. Three *Tp53/Rb1/Uhrf1* TKO mice did not acquire tumors well beyond the average of the rest of their study group (>69 weeks; Fig. 6B^†^). FDG-PET/microCT scans in these three mice revealed no detectable tumors (Supplementary Fig. 8E), which was confirmed upon autopsy.

Since UHRF1 is also overexpressed in *Tp53* cKO tumors (Fig. 6A and Supplementary Fig. 8B), we examined the effect of *Uhrf1* loss in *Tp53/Uhrf1* DKO compared to *Tp53* cKO mice. We observed a significant increase in median survival of *Tp53/Uhrf1* DKO mice, compared to *Tp53* cKO (*P* = 0.0007, Gehan–Breslow–Wilcoxon test), but not in overall survival (*P* = 0.1189, Mantel–Cox test; Fig. 6B). Interestingly, we found that all *Tp53* cKO mice with early morbidity (<50 weeks) presented tumors with increased *Uhrf1* mRNA levels, suggesting spontaneous RB pathway inactivation (Supplementary Fig. 8F). High *Uhrf1* mRNA expression correlated with increased *Cdk4* expression (Supplementary Fig. 8G). No correlation was found with the expression of other RB regulators, including *p16* and *Cdk6* (Supplementary Fig. 8H, I). No significant changes on *Rb1* expression were observed between these groups (Supplementary Fig. 8J). Histopathology analysis of tumors from the four mouse models analyzed linked *Uhrf1* loss with lower mitotic index and decreased anaplasia (Supplementary Fig. 9 and Supplementary Table 2).

Most remarkably, in line with our in vitro observations, *Uhrf1* loss resulted in a significant reduction of pulmonary metastases, with only 22.6% of *Tp53/Rb1/Uhrf1* TKO mice presenting lung metastases compared to 52.4% of *Tp53/Rb1* DKO mice. A smaller number of metastatic nodules was also observed in the *Tp53/Rb1/Uhrf1* TKO mice that developed lung metastases (Fig. 6C).

To determine whether *Uhrf1* contributes to tumor promotion or tumor progression, we performed periodic FDG-PET/microCT scans on *Tp53/Rb1* DKO and *Tp53/Rb1/Uhrf1* TKO mice to determine the age at which tumors are detectable (Supplementary Fig. 8A). In *Tp53/Rb1/Uhrf1* TKO mice, tumors were detected significantly later than in *Tp53/Rb1* DKO mice (*P* = 0.0362; Fig. 6C). All test subjects reached humane end-of-study within 3 weeks of tumor detection. These data suggest that *Uhrf1* plays a pivotal role in the early developmental process of osteosarcoma promotion following the loss of *Rb1*.

## Discussion

*RB1* genetic alterations are associated with increased mortality, metastasis, and poor response to chemotherapy in osteosarcoma [5–9, 37, 38]. However, the precise mechanism through which this occurs remains to be elucidated. Studies from retinoblastoma, a cancer initiated by biallelic inactivation of the *RB1* gene, defined a central role of RB in epigenetic control and a key role of RB/E2F-regulated chromatin remodelers in tumorigenesis [10, 39, 40]. Among others, UHRF1 was identified as a potential critical regulator of tumor initiation and progression following RB pathway inactivation [10, 13–16]. Here, we identified UHRF1 overexpression as directly correlated with increased osteosarcoma malignancy and metastatic disease.

UHRF1 expression is positively correlated with cell proliferation and cell mobility in both normal and malignant cells in vitro [10, 25–28]. Here, we show that UHRF1 KO decreases clonogenicity and tumor growth both in vitro and in vivo. UHRF1 also has a clear effect on migration. UHRF1 overexpression induced migration and invasion of normal MSCs and targeting UHRF1 drastically reduced migration and invasion of osteosarcoma cell lines. It is important to note that although *RB1*-null status has been clinically associated with poor disease outlook, the *RB1*-null cell line, SaOS-2, does not exhibit higher aggressiveness in comparison to other cell lines. This could be explained by the limitation of utilizing cell lines, in which loss of clinical representation is commonly seen.

This study also presents novel roles of UHRF1 in controlling exosome formation and angiogenesis. Exosomes and their cargo are implicated in osteosarcoma metastasis [41]. Our study shows that UHRF1 alters the expression of exosomal components and their protein cargo composition. We identified uPA as a critical factor in UHRF1-mediated migration, in line with a previous study describing the uPA/uPAR axis as a metastatic driver in osteosarcoma [42]. uPA is a serine protease associated with cell migration and a known cargo protein in osteosarcoma-secreted exosomes [30]. Upon plasminogen conversion into plasmin, uPA triggers a proteolytic cascade leading to degradation of extracellular matrix, an event necessary for angiogenesis and metastasis. While our results suggest that targeting UHRF1 might result in a more robust therapeutic outcome, the observation that cell migration can be reversed by treatment with uPA inhibitors like amiloride [43] and BC11 hydrobromide, provides therapeutic alternatives that can be further explored (Fig. 5D).

Arising from the observation that UHRF1 KO xenografts presented with reduced tumor vascularization, we identified a new role of UHRF1 in the induction of angiogenesis. Mechanistically, we propose a UHRF1-AMPK-SEMA3E axis where SEMA3E repels endothelial sprouting leading to decreased angiogenesis (Fig. 5D). This is supported by the recent report showing that UHRF1 suppresses AMPK activity in the nucleus by recruiting PP2A to dephosphorylate AMPK [32] and previous findings showing that AMPK activation induces *SEMA3E* expression [33]. SEMA3E is known to act as a repulsive factor for endothelial cells that express plexin-D1, resulting in decreased neoangiogenesis and reduced tumor growth [31].

Our group is the first to model the critical role of UHRF1 in osteosarcomagenesis, with *Uhrf1* KO resulting in a dramatic increase in overall survival and a decrease in metastases, particularly in *Rb1*-null osteosarcoma. The survival and rate of metastasis of *Tp53*/*Rb1*/*Uhrf1* TKO closely resembled that of *Tp53* cKO mice, indicating that UHRF1 is a major driver of *Rb1* loss-associated malignancy. Interestingly, our data also indicate that the increased survival in *Tp53*/*Rb1*/*Uhrf1* TKO mice is largely a result of delayed tumor promotion, with negligible differences in tumor growth rates once the tumors have been detected. However, decreased growth rates were observed when UHRF1 KO was induced in established tumors. This distinct observation suggests that there might be compensatory pathways arising in the developmental tumor models. Further analyses on tumors arising from these developmental osteosarcoma models might provide insight on potential compensatory mechanisms of *Uhrf1* loss which may be relevant for predicting mechanisms of resistance for future UHRF1-targeted therapeutics.

While targeting *Uhrf1* in developmental mouse models showed drastic improvement in survival in *Rb1*-null tumors over *Rb1*-wild-type, such distinctions were not observed in UHRF1 overexpressing human osteosarcoma cell lines. Both *RB1*-null and *RB1*-wild-type osteosarcoma cell lines appeared to equally benefit from targeting UHRF1. It is possible that the timing of UHRF1 overexpression is crucial for the resulting poor prognosis. Early *Rb1* loss would result in immediate UHRF1 overexpression which quickly promotes tumorigenesis compared to tumors that eventually inactivate the RB/E2F pathway through alternative mechanisms. Supporting this theory, *Tp53/Uhrf1* DKO mice showed improvement in survival compared to a subpopulation of *Tp53* cKO mice that exhibited characteristics of RB/E2F pathway inactivation and therefore presented early morbidity. Alternatively, the RB/E2F complex is known not only for its role in direct transcriptional repression, but also as epigenetic modifiers through the recruitment of chromatin remodelers [39, 44–47]. Two UHRF1 domains, PHD and RING, contain LXCXE RB-binding motifs [48]. Unpublished data from our group show UHRF1 preferentially binds to hyperphosphorylated RB. Thus, while the transcriptional regulation of UHRF1 is aberrant by means of RB/E2F pathway inactivation, *RB1*-wild-type osteosarcoma cells may preserve protein–protein interactions between RB and UHRF1. As a complex, RB and UHRF1 may exert regulation on downstream targets, including UHRF1 itself. The possible loss of this layer of regulation in *RB1-*null tumors could account for the drastic difference in clinical prognosis, supporting the view that the inactivation of RB by phosphorylation is not functionally equivalent to the mutation of the *RB1* gene [49].

This study supports targeting UHRF1 as a novel therapeutic strategy and provides alternative therapeutic interventions along the RB-UHRF1 axis for osteosarcoma treatment. Targeting UHRF1 holds great potential in overcoming the highly metastatic characteristic of osteosarcoma, the main reason why the survival rate has remained stagnant in past decades. We are the first to model the effect of UHRF1 throughout development and showed minimal on-target toxicity, as opposed to various chemotherapeutic agents known to affect bone homeostasis [50]. Moreover, the degree to which UHRF1 loss reverted the increased malignancy upon *Rb1* loss suggests that the benefit of UHRF1 targeting could go beyond the scope of osteosarcoma by improving current treatment paradigms of other cancers harboring RB/E2F pathway inactivation.

## Materials and methods

### Xenografts and mouse models

The orthotopic xenografts used in this study (all *TP53* mutant): SJSO010930 (PDX1; *RB1* mutant), SJOS001112 (PDX2; *RB1* mutant), SJOS001107 (PDX3; *RB1* wt), SJOS001105 (PDX4; *RB1* wt), and SJOS001121 (PDX5; *RB1* mutant) were obtained from the Childhood Solid Tumor Network [51]. Athymic nude (NU/J) and NOD *scid* gamma (NSG) mice were obtained from The Jackson Laboratories. *p53*^*lox*^ and *Rb1*^lox^ mice were obtained from the Mouse Models of Human Cancer Consortium at the National Cancer Institute; *Osx-cre* mice from The Jackson Laboratory. *Uhrf1* cKO mice obtained from EMMA were backcrossed to flipasse (Flp) mice to remove the neo-cassette (tm1c conversion) and backcrossed to C57BL/6N mice for Flp removal. Mice were monitored weekly for signs of osteosarcoma. Moribund status defined as the point when tumors had reached 10% body weight or induced mouse paralysis. Experiments include animals from both sexes. The University of California Irvine Institutional Animal Care and Use Committee approved all animal procedures.

### Cell culture

Osteosarcoma cell lines 143B, SJSA-1, SaOS-2, and U-2 OS, as well as bone marrow-derived MSCs and HEK293T cells were acquired from ATCC and maintained according to their specifications. Human umbilical vein endothelial cells (HUVEC) expressing blue fluorescent protein were cultured in EGM-2 (Lonza CC-3162). Normal human lung fibroblasts (hLF) were routinely cultured in DMEM + 10% FBS. All cells were routinely checked for mycoplasma contamination, and stocks were STR profiled at the time of freezing.

Cell lines were analyzed as clones (*n* = 3) or as pools examined within 2–3 weeks after being generated.

### Western blotting

Western blots were performed as described [52] using the following antibodies and dilutions: 1:1000 Uhrf1 (sc-373750, Santa Cruz Biotechnology), 1:1000 Rb (9313T, Cell Signaling), actin (A1978, Sigma), E2F1 (3742S, Cell Signaling), E2F2 (sc-9967, Santa Cruz Biotechnology), E2F3 (MA5-11319, Nalgene Nunc); 1:1000 SEMA3E (PA547469, Thermo Scientific); 1:1000 AMPK (5831S, Cell Signaling); 1:1000 pAMPK (ab133448, Abcam). Secondary antibodies were diluted 1:1000 (PI-1000, PI-2000 or PI-9500, Vector Laboratories). Bands were visualized using chemiluminescence (SuperSignal West Pico Chemiluminescent Substrate, Thermo Scientific). Band intensities were analyzed using ImageJ software.

### In situ hybridization

Formalin-fixed, paraffin-embedded (FFPE) slides were incubated at 60 °C for 1 h for deparaffinization, washed twice in xylene and twice in 100% ethanol, 5 min each at RT before air-drying. RNAscope was performed following the manufacturer’s protocol. Human and mouse UHRF1-specific probes were customized by Bio-Techne. RNAscope^®^ Positive Control Probe- Hs-PPIB/Mm-PP1B and RNAscope^®^ Negative Control Probe- DapB were used. OpalTM 570 fluorophore was used at a 1:1000 dilution. Slides were mounted with ProLong Gold Antifade Mountant.

### Real-time RT-PCR (qPCR)

We performed qPCR as described [52]. Primers were designed using IDT Real-Time PCR tool (Integrated DNA Technologies). Reaction was carried out using 7500 Real-Time PCR system (Applied Biosystems). Data were normalized to those obtained with endogenous control 18 S mRNA and analyzed using ΔΔCt method. Primer sequence for PCR amplification are as follows: *UHRF1* (5’-GCTGTTGATGTTTCTGGTGTC-3’; 5’-TGCTGTCAGGAAGATGCTTG-3’), *PLAU* (5’-GAGCAGAGACACTAACGACTTC-3’; 5’-CTCACACTTACACTCACAGCC-3’), *SEMA3E* (5’-CTGGCTCGAGACCCTTACTG-3’; 5’- CAAAGCATCCCCAACAAACT-3’), *HAS2* (5’-TCCATGTTTTGACGTTTGCAG-3’; 5’-AGCAGTGATATGTCTCCTTTGG-3’), *LAMC2* (5’-CACCATACTCCTTGCTTCCTG-3’; 5’-GTGCAGTTTGTCTTTCCATCC-3’), *18* *S* (5’-GTAACCCGTTGAACCCCATT-3’; 5’-CCATCCAATCGGTAGTAGCG-3’).

### Lentivirus production and transduction

Lentiviral particles were produced as previously described [52]. plentiCRISPRv2 (GenScript) with UHRF1 gRNA (gRNA sequence: TCAATGAGTACGTCGATGCT), inducible CRISPR/Cas9 plasmid TLCV2 (Addgene plasmid #87360) with UHRF1 gRNA (gRNA1 sequence: CGCCGACACCATGTGGATCC and gRNA2 sequence: ACACCATGTGGATCCAGGTT) were used.

### Clonogenic assay

Colony formation assay was performed as previously described [52].

### Subcutaneous injection

In total, 2 × 10^6^ cells were resuspended in 100 μl PBS and injected subcutaneously into the flank region of mice. Tumors were caliper-measured and collected 3 weeks post injection. For doxycycline induction, 2 mg/ml doxycycline hyclate supplemented water was administered ad libitum. 10 mg/ml sucrose was added to doxycycline-supplemented water to increase consumption.

### Intrafemoral injection

Cell-PBS mixture (4 × 10^6^ cells/ml) was mixed with an equal volume of 7.7 mg/ml Matrigel (Corning, 356230) to achieve a final concentration of 2 × 10^6^ cells/ml. Cell mixture, needle, and 50-µl syringe (Hamilton, 80920) were left in ice until ready for injection. NSG mice were laid down in dorsal recumbency and the injection area was shaved and prepared. The right femur was stabilized and positioned perpendicular to the hood. A 25 G needle was inserted into the femur through the kneecap with a twist motion to create the space for injection later. A new 25 G needle loaded with 10 µL cell–Matrigel mixture was inserted back to the femur. The cell mixture was slowly injected to prevent leakage. Mice were monitored weekly after injection. Primary tumors, lungs, and the injected femurs were collected and used for analysis.

### RNA sequencing

RNA sequencing was performed as described [53]. All sequencing data discussed in this publication have been deposited in NCBI’s Gene Expression Omnibus [54] and are accessible through GEO Series accession number GSE144418.

### Scratch-wound-healing assay

The scratch-wound assay was performed as previously described [52]. Two hours prior scratch, cells were treated with 5 µg/ml mitomycin C (S8146, Sigma) to control for proliferation differences. Amiloride was used at 150 µM, BC11 hydrobromide at 16.5 µM, GW4869 at 10 µM, which were supplemented in growth medium and given to the cells at the 0 h time point. Chlorpromazine (CPZ) was used at 20 µM for 30 min prior to scratch. For experiments using conditioned media, serum-free media was used and exposed to cells for 24 h.

### Transwell invasion assay

In all, 8-µm pore PET membranes (353097, Corning) were coated with Matrigel (25 µg/insert). In all, 2 × 10^4^ cells were seeded onto the membrane in 100 µl of appropriate cell culture medium and placed into the incubator for 2 h at 37 °C with 5% CO_2_ to allow cell attachment. After cells have attached, 100 µl of media were added to the inner chamber, amiloride (150 µM final concentration) or BC11 hydrobromide (16.4 µM final concentration) or doxycycline (1 µg/ml final concentration) were supplemented at this time. In total, 600 µl of appropriate cell culture medium supplemented with 100 ng/ml fibroblast growth factor (FGF) was added to the outer chamber. Cells were incubated for 16 h at 37 °C with 5% CO_2_. The cell culture medium was aspirated, and cotton swabs were used to remove the remaining cells inside the inner chamber. Cells at the bottom side of the insert were then fixed and stained with 0.5% crystal violet for 2 h. Excess dye was washed out with tap water. Images of five different fields within each insert were taken for analysis by cell count.

### Tail vein injection

In total, 2 × 10^6^ cells were resuspended in 200 µl PBS and injected intravenously through the tail vein. Lungs were collected 3 weeks after injection and fixed in 4% formaldehyde for histological analysis.

### 3D fibrin gel angiogenesis assay

Fibrin gel angiogenesis assays were performed as previously described [55]. Briefly, HUVECs were coated onto Cytodex 3 microcarrier beads (GE 17048501) at a concentration of 150 cells/bead for 4 h and allowed to adhere overnight. HUVEC-coated beads were then resuspended in a 2.5 mg/ml fibrinogen solution (MP Biomedicals) at a concentration of 250 beads/ml. Gels were formed by adding 500 µl of the fibrinogen/bead suspension to each well of a 24-well plate containing 0.5 U of thrombin (Sigma-Aldrich). Once gels were clotted, 1 ml of EMG2 conditioned media from osteosarcoma VC or KO cells or containing 20,000–50,000 hLFs (as assay control) was added to each well. Assays were quantified between days 6 by live-culture imaging using bright-field and fluorescence microscopy. Thirty beads per condition were quantified per experiment.

### Statistical analysis

All graphing and statistical analyses were performed using GraphPad Prism (version 9 for Mac, GraphPad Software, San Diego, CA, USA). All microscopy images were randomly taken from different areas. Details of sample size (*n*), statistical test, and *P* value applied for each experiment were indicated in the figure legends. Results are presented as median ± SD. The statistical significance of differences between the two groups was assessed by Student’s *t* test. *P* values < 0.05 were considered statistically significant. Survival curves were analyzed using log-rank (Mantel–Cox) and Gehan–Breslow–Wilcoxon tests. Animal numbers were calculated from pilot studies and biostatistical calculation of a minimum of 75% power at 0.05 significance. Animals euthanized for non-study-related pre-established criteria were excluded from the analysis. Phenotypic measurements were scored blinded.

## Supplementary information

Supplemental Figure 1

Supplemental Figure 2

Supplemental Figure 3

Supplemental Figure 4

Supplemental Figure 5

Supplemental Figure 6

Supplemental Figure 7

Supplemental Figure 8

Supplemental Figure 9

Supplemental Figure 10

Supplemental Figure Legends

Supplemental Table 1

Supplemental Table 2

## Data Availability

All data supporting the findings of this study are available within the article and its supplementary information files and from the corresponding author upon reasonable request.
