# Supplementary material for: UHRF1 overexpression promotes osteosarcoma metastasis through altered exosome production and AMPK/SEMA3E suppression
Source: Oncogenesis. 2022 Sep 6;11(1):51. doi: 10.1038/s41389-022-00430-6 (PMC9448786; doi:10.1038/s41389-022-00430-6)
Supplement: Supplementary file 11 — Supplemental Figure Legends [file 41389_2022_430_MOESM11_ESM.docx]

**Supplemental Figure Legends**

**Supplemental Figure 1.** (A-B) The Cancer Genome Atlas data of (A) RNA-Seq by expectation-maximization (RSEM) values from 263 human sarcoma (SARC) tumor samples compared to 2 normal tissue (17.9-fold increase), 1100 breast invasive carcinoma (BRCA) compared to 112 normal (13.5-fold), 517 lung adenocarcinoma (LUAD) compared to 59 normal (13.7-fold) show increased *UHRF1* expression in tumors with frequent RB pathway alterations but not in 498 prostate adenocarcinoma (PRAD) tumors compared 52 normal (1.84-fold) were RB pathway is normally unaltered. Tumor samples (red) compared to normal samples (blue). (B) Effect of *UHRF1* expression level on SARC patient survival comparing high UHRF1 expression (red; n=65) to low/medium UHRF1 expression (blue; n=194). (C) Representative fluorescent images in 2X (top) and 20X (bottom) magnification of *in situ* hybridizations using RNAscope scored as low or high based on signal intensity for *UHRF1* mRNA. Scale bar = 100 µm. (D) Quantification of the percentage of low and high signal score acquired from RNAscope in stage IA (n=6), IB (n=16), and IIB (n=16) OS. Unfortunately, the limited number of stage III and stage IV samples available restricted analysis of metastatic cases. (E-F) qPCR analysis of *UHRF1* mRNA in (E) human OS cell lines (143B, SJSA-1, SaOS-2 and U-2 OS) and (F) patient-derived xenografts (PDX1-5). All data are mean ± SD normalized to MSC (n=3). (G-H) Representative Western blot analysis of RB protein presence in (G) human OS cell lines and (H) PDXs. β-actin was used as loading control. ** p < 0.01, *** p < 0.001 by unpaired two-tailed *t* test.

**Supplemental Figure 2.** (A) Chromatin immunoprecipitation (ChIP) assay reveals enrichment of E2F1 at the three putative binding motifs (M1-3) within *UHRF1* promoter, as predicted by MotifMap. (B-E) Western blot analysis of UHRF1 protein in OS cells transduced with lentivirus containing shRNA against (B) E2F1 (shE2F1) show absence of change in UHRF1 expression upon E2F1 knockdown compared to scramble (shScrmbl), (C) E2F1 and E2F2 (shE2F1/2) combined knockdown also does not alter UHRF1 expression, however (D) E2F1 and E2F3 (shE2F1/3) combined knockdown reduces UHRF1 expression compared to scrambled control. (E) E2F3 (shE2F3) knockdown alone is not sufficient to alter UHRF1 expression. β-actin was used as loading control. Band intensities were quantified by densitometry and normalized to scrambled control. (F) qPCR analysis of *UHRF1* mRNA expression using increasing doses of palbociclib. Data are mean ± SD normalized to DMSO control (n=3). (G) Western blot analysis of UHRF1 protein level in OS cells treated with increasing doses of palbociclib. (H-I) qPCR analysis of (H) *CDK4* and *CDK6* and (I) *INK4A* mRNA expression. Data are mean ± SD normalized to MSC1. *p < 0.05, ** p < 0.01, *** p < 0.001 by unpaired two-tailed *t* test.

**Supplemental Figure 3.** (A) Growth curves of UHRF1 knockout cells (KO, red) compared to control (VC, black), population doubling time indicated in parentheses. (B) Representative images of cells labelled with Click-iT EdU (green) or immunostained with UHRF1 (red). Nuclei were counterstained with DAPI (blue). (C) Quantification of EdU signal from immunocytochemistry probing UHRF1 and EdU in VC and UHRF1 KO human OS cell lines. ns=not significant; ** p < 0.01, *** p < 0.001 by unpaired two-tailed *t* test.

**Supplemental Figure 4.**  (A-B) Quantification of the SJSA-1 iCRISPR VC (iVC, n=5) and iCRISPR KO gRNA2 (iKO, n=5) tumor volume over time (A) and final tumor volume (B) for each of the replicates. (C-D) Quantification of the 143B iCRISPR VC (iVC, n=5) and iCRISPR KO (iKO, n=5) tumor volume over time (C) and final tumor volume (D) for each of the replicates. Data are mean ± SD. *p < 0.05, ** p < 0.01, *** p < 0.001 by paired two-tailed *t* test. (E) Western blot verification of UHRF1 protein levels in SJSA-1 intrafemoral xenograft tumors for VC and UHRF1 KO. β-actin was used as loading control.

**Supplemental Figure 5.** (A) Plasmid map of the doxycycline inducible UHRF1 overexpression system. Tet Response Element (TRE) is activated by doxycycline binding to the reverse tetracycline-controlled transactivator (rTA), allowing transcription of GFP and the pCW57-promoter driven human UHRF1 cDNA. (B) Western blot detection of UHRF1 in MSCs transduced with vector control (pCW57-VC) or UHRF1 overexpression (pCW57-UHRF1) plasmid, treated with DMSO (-) or doxycycline (+). (C) Scratch-wound assay in MSC culture comparing wound closure of control (pCW57-VC) and UHRF1 overexpressed (pCW57-UHRF1) cells over 8 h span. White dashed lines represent wound edge. (D) Histogram quantification of distance (pixels) migrated for doxycycline non-induced (black) and induced (gray) pCW57-VC and pCW57-UHRF1. Each data point is mean ± s.d. of ten measurements in triplicate samples. (E) Representative images from Transwell assays with cells stained with crystal violet comparing levels of invasion between doxycycline non-induced and induced pCW57-VC and pCW57-UHRF1 MSC. (F) Quantification of the number of cells invaded across the Transwell membrane. Each data point is mean ± s.d. of triplicate samples. (G) Western blot detection of UHRF1 in OS wild-type and OS UHRF1 KO cells transduced with the inducible UHRF1 overexpression (pCW57-UHRF1) plasmid, treated with DMSO (-) or doxycycline (+). Relative quantification of the UHRF1 levels are shown in the bottom, normalized to wild-type levels. (H) Histogram quantification of distance (pixels) migrated for doxycycline non-induced (black) and induced (gray) pCW57-UHRF1. Each data point is mean ± s.d. of ten measurements in triplicate samples. *p < 0.05, ** p < 0.01, by unpaired two-tailed *t* test.For all graphs: ns=not significant, ** p < 0.01, *** p < 0.001, by unpaired two-tailed *t* test.

**Supplemental Figure 6.** (A) Representative H&E staining from lung sections collected from mice injected intravenously with VC or UHRF1 KO SJSA-1 cells. Pulmonary nodules were quantified by (B) the number of tumor nodules found per lung and by (C) the average tumor size per lung. Solid line represents the average from n=7 for VC and n=6 for KO. (D) Cartoon representation for doxycycline-inducible *in vivo* knockout for tail-vein-injected osteosarcoma cell lines carrying a non-targeting sgRNA (iCRISPR VC; iVC) or UHRF1 sgRNA (iCRISPR KO; iKO) to assess lung metastases. Pulmonary nodules were quantified by (E) the number of tumor nodules found per lung and by (F) the average tumor size per lung. Solid line represents the average from five replicates for each condition. (G) Representative histological images of F4/80 macrophage marker expression in intrafemoral tumors derived from VC and UHRF1 KO SJSA-1 cells, 5-weeks after injection and (H) quantification of the mean F4/80 signal intensity per mm^2^. Solid line represents mean from n=4. For all graphs: ns, not significant, * p< 0.05, ** p < 0.01, by unpaired two-tailed *t* test.

**Supplemental Figure 7.** (A) Dot blot detection of 5-methyl cytosine signal in non-targeting vector control (VC) and UHRF1 KO (KO) OS cells, comparing global methylation levels. (B) Heatmap for differentially methylated regions generated from RRBS across the genome of VC and KO OS cells. (C) Chromatin landscape generated from ATAC-seq showing peaks of chromatin accessibility across the genome of VC and KO OS cells. (D) Principal component analysis (PCA) plot generated from RNA-seq displaying gene expression profile in VC and KO OS cells. (E) qPCR analysis confirming expression changes of migration related genes in individual cell lines upon UHRF1 loss, fold change of mRNA level normalized to vector control (gray), compared with average fold change from RNA-seq analysis (black).

**Supplemental Figure 8.** (A) Representative images from Transwell assays with cells stained with crystal violet comparing levels of invasion between doxycycline induced pCW57-VC and pCW57-UHRF1 MSC treated with DMSO or 150 µM amiloride. (B) Quantification of the number of MSC invaded across the Transwell membrane from experiment shown in G. Each data point is mean ± s.d. of triplicate samples. ns=not significant, ** p < 0.01

**Supplemental Figure 9.** (A) Representative micro-CT (top) and micro-PET (bottom) scans of OS that arises in a mouse femur used for determining the age at which tumors can be first detected (red arrow). (B) Western blot detection of UHRF1 in tumors from genetically engineered *p53* cKO and *p53/Rb1* DKO OS mice, β-actin was used as loading control. (C) Micro-CT scan of hind limb from wildtype (w.t.) and preosteoblastic *Uhrf1* knockout (*Uhrf1* KO) mice. (D) Bone density analysis of prime OS locations in Hounsfield units (HU). Each data point is mean ± s.d. of n=4 samples. (E) FDG-PET/microCT scans from the three *Tp53/Rb1/Uhrf1* TKO mice that had not acquired tumors well beyond the average of the rest of their study group (> 69 weeks) show no signs of detectable tumors. (F-J) qPCR analysis of (F) *Uhrf1*, (G) Cdk4, (H) *p16*, (I) *Cdk6*, and (J) *RB1* mRNA expression in osteosarcoma mouse tumors from the 70% lower age to end-of-study (young; n=8) compared to the older 30% (old; n=3) mice. One tumor with undetectable *p16* expression was identified from a mouse with early morbidity. Data are mean ± SD normalized to mouse MSC.

**Supplemental Figure 10.** Representative H&E staining from OS tumor sections collected from genetically engineered osteosarcoma mouse models subjected to pathology analysis: *Tp53/Rb1* DKO: *Osx-Cre; p53^lox/lox^; Rb1^lox/lox^*, *Tp53*/*Rb1*/*Uhrf1* TKO: *Osx-Cre; p53^lox/lox^; Rb1^lox/lox^* ;*Uhrf1^lox/lox^* , *Tp53* cKO: *Osx-Cre; p53^lox/lox^; Rb1^lox/lox^* and *Tp53*/*Uhrf1* DKO: *Osx-Cre; p53^lox/lox^; Uhrf1^lox/lox^* . Scale bar = 100 μm.

**Supplemental Materials and Methods**

***In situ* hybridization.** Osteosarcoma tissue microarrays were purchased from US Biomax, Inc (OS804c). 6 stage IA (T1N0M0), 16 stage IB (T2N0M0), 1 stage IIA (T1N0M0), 16 stage IIB (T2N0M0) and 1 stage IVB (T2N1M0) tumors are represented in the array. Stage I tumors are low grade and stage II and IV are high grade. Extent of the primary tumor (T) is classified as intercompartmental (T1) or extracompartmental (T2). Tumors metastasized to nearby lymph nodes are denoted by N1, but no distant organ metastases (M0) are represented in the array. Formalin-fixed, paraffin-embedded (FFPE) slides were incubated at 60°C for 1 h for deparaffinization, washed twice in xylene and twice in 100% ethanol, 5 min each at RT before air-drying. RNAscope was performed following manufacturer’s protocol. Human UHRF1-specific probes were customized by Bio-Techne. RNAscope® Positive Control Probe- Hs-PPIB and RNAscope® Negative Control Probe- DapB were used. OpalTM 570 fluorophore was used at a 1:1000 dilution. Slides were mounted with ProLong Gold Antifade Mountant.

**Chromatin immunoprecipitation**

ChIP assays were performed as previously described [17]. ChIP DNA was analyzed by qPCR with SYBR Green (Bio-Rad) in ABI-7500 (Applied Biosystems) using the following primers: Forward: 5′- CACCCTCTTTCTCGCTTCC-3′; Reverse 5′-TGCCAGCTGCTCTGATTT-3′ (spanning M1); Forward: 5′- CCACATTCCCTCGCAGTATTTA-3′; Reverse 5′-CCCTGAACTCTTAAGTCCAAGTC-3′ (in close proximity with M2, 3). The antibodies used were anti-E2F1 (3742; Cell Signaling) and rabbit IgG (sc-2027, Santa Cruz Biotechnologies).

**5mC DNA dot blot**

Genomic DNA was extracted from cells (Wizard SV genomic DNA purification Kit). Purified DNA was quantified, sonicated, denatured and transferred onto Nylon membrane (RPN303B, GE Healthcare) according to the Cell Signaling DNA Dot Blot Protocol. 5-methylcytosine (5-mC) Ab (#28692, Cell Signaling) was used to detect global DNA methylation of the samples. Methylene blue staining was used to verify equal DNA loading across samples.

**Chromatin profiling**

Approximately 50,000 cells were harvested for ATAC-seq for each replicate. ATAC-seq was performed as previously described [23]. ATAC-seq libraries were sequenced with the Illumina HiSeq4000 using 100bp paired-end single indexed run. Raw reads were first QCed (*FASTQC*) and quality and adapter trimmed using *Trimmomatic*. Trimmed reads were then aligned to hg19 build of the human genome using Bowtie2 (v2.2.5) with alignment parameters: bowtie2 -X 2000– local–dovetail. Potential PCR duplicate reads were removed using *MarkDuplicates* from the *Picard* tools. Peaks were called in each sample using *MACS2* and further filtered using the ENCODE consensus blacklist regions (http:// hgdownload.cse.ucsc.edu/goldenPath/hg19/encodeDCC/wgEncodeMapability/). Differential peaks were identified using R package *diffbind* across the consolidated peak sets and metrics such as adjusted p values were reported.

**FDG-PET/microCT scan.** Overnight-fasted mice were injected with 0.1-0.5 mCi of ^18^F-FDG in sterile saline (0.05-0.2 ml) intraperitoneally (i.p.) 60 min prior to imaging. Imaging was performed on anesthetized animals laid in supine position. Scanning data was acquired in full list mode and sorted into a single frame, 3 dimensional sinogram, which was rebinned using a Fourier rebinning algorithm. The images were reconstructed using 2-dimensional filter back projection using a Hanning Filter with a Nyquist cut off at 0.5 and corrected for attenuation using the Co-57 attenuation scan data. Analysis of PET was conducted using PMOD 3.0 and IRW software. The PET data was co-registered to the CT template for drawing regions-of-interest (ROI). The ROI data was converted to standard uptake value (SUV) of ^18^F-FDG.

**References:**

Canver, M.C., Smith, E.C., Sher, F., Pinello, L., Sanjana, N.E., Shalem, O., Chen, D.D., Schupp, P.G., Vinjamur, D.S., Garcia, S.P.*, et al.* (2015). BCL11A enhancer dissection by Cas9-mediated in situ saturating mutagenesis. Nature *527*, 192-197.

Chen, C.W., Koche, R.P., Sinha, A.U., Deshpande, A.J., Zhu, N., Eng, R., Doench, J.G., Xu, H., Chu, S.H., Qi, J.*, et al.* (2015). DOT1L inhibits SIRT1-mediated epigenetic silencing to maintain leukemic gene expression in MLL-rearranged leukemia. Nat Med *21*, 335-343.

Doench, J.G., Fusi, N., Sullender, M., Hegde, M., Vaimberg, E.W., Donovan, K.F., Smith, I., Tothova, Z., Wilen, C., Orchard, R.*, et al.* (2016). Optimized sgRNA design to maximize activity and minimize off-target effects of CRISPR-Cas9. Nat Biotechnol *34*, 184-191.

Langmead, B., Trapnell, C., Pop, M., and Salzberg, S.L. (2009). Ultrafast and memory-efficient alignment of short DNA sequences to the human genome. Genome Biol *10*, R25.

Robinson, M.D., McCarthy, D.J., and Smyth, G.K. (2010). edgeR: a Bioconductor package for differential expression analysis of digital gene expression data. Bioinformatics *26*, 139-140.

Uckelmann, H.J., Kim, S.M., Wong, E.M., Hatton, C., Giovinazzo, H., Gadrey, J.Y., Krivtsov, A.V., Rucker, F.G., Dohner, K., McGeehan, G.M.*, et al.* (2020). Therapeutic targeting of preleukemia cells in a mouse model of NPM1 mutant acute myeloid leukemia. Science *367*, 586-590.
