# Supplementary material for: UHRF1 overexpression promotes osteosarcoma metastasis through altered exosome production and AMPK/SEMA3E suppression
Source: Oncogenesis. 2022 Sep 6;11(1):51. doi: 10.1038/s41389-022-00430-6 (PMC9448786; doi:10.1038/s41389-022-00430-6)
Supplement: Supplementary file 12 — Supplemental Table 1 [file 41389_2022_430_MOESM12_ESM.pdf]

Supplemental Table 1. ATAC-seq results p<0.05

	seqnames	start	end	Conc_VO	Conc_UHRF	Fold	p.value
7763	chr5	38147946	38148446	3.36	6.07	-2.71	0.000352
3317	chr15	33406160	33406660	4.57	6.72	-2.14	0.0129
7767	chr5	38756061	38756561	4.92	6.76	-1.85	0.0249
4377	chr17	68223297	68223797	4.56	6.3	-1.74	0.015
8608	chr6	44084322	44084822	4.31	5.99	-1.67	0.0285
5989	chr20	23125981	23126481	4.16	5.77	-1.62	0.0167
5965	chr20	17519102	17519602	3.91	5.45	-1.54	0.0138
5084	chr2	23637633	23638133	5.18	6.5	-1.31	0.0293
5213	chr2	49056145	49056645	4.48	5.78	-1.3	0.0492
4829	chr19	30369822	30370322	5.99	7.24	-1.25	0.034
6767	chr3	46688121	46688621	4.46	5.66	-1.2	0.0402
2655	chr13	36705232	36705732	6.35	5.38	0.97	0.0439
9982	chr9	4741018	4741518	7.73	6.66	1.07	0.0374
1176	chr10	63422386	63422886	6.64	5.38	1.26	0.0303
9071	chr7	25990604	25991104	6.38	4.93	1.45	0.0352
5460	chr2	125593939	125594439	6.54	4.93	1.61	0.0328
