# Supplementary material for: UHRF1 overexpression promotes osteosarcoma metastasis through altered exosome production and AMPK/SEMA3E suppression
Source: Oncogenesis. 2022 Sep 6;11(1):51. doi: 10.1038/s41389-022-00430-6 (PMC9448786; doi:10.1038/s41389-022-00430-6)
Supplement: Supplementary file 13 — Supplemental Table 2 [file 41389_2022_430_MOESM13_ESM.pdf]

Supplemental Table 2. Pathology analysis of mouse osteosarcoma strains

Genotype	Tumor ID	Malignant osteoid formation	Tumor nuclear size	Anaplasia/Significant pleomorphism	Mitotic activity	Necrosis
**p53 cKO**	OXTP75	No	Medium to large sized with epithelioid features	Focal	3/10 HPF	Yes, small focus
	OXTP89	No	Medium to large sized	Yes	3/10 HPF	No
	OXTP73	Yes, abundant	Medium to large sized	No	1/10 HPF	No
**p53/Rb1 DKO**	OTP223	Yes, rare	Medium to large sized with epithelioid features	Yes	4/10 HPF	No
	OTP196	Yes, abundant	Medium sized, frequent MNT cells	No	1-2/10 HPF	No
	OTP241	No	Small to medium sized	Yes	10/10 HPF	Yes
**p53/Uhrf1 DKO**	UOTX414	Yes, abundant	Small sized	No	0/10 HPF	No
	UOTX373	Yes, abundant	small to Medium sized, rare MNT cells	No	0/10 HPF	No
	UOTX384	Yes, abundant	small to Medium sized, rare MNT cells	No	1/10 HPF	No
**p53/Rb1/Uhrf1 TKO**	UOT259	Yes, abundant	Medium sized, frequent MNT cells	No	1/10 HPF	No
	UOT318	Yes, abundant	Medium sized, occasional MNT cells	No	1/10 HPF	No
	UOT308	Yes, abundant	Medium sized, occasional MNT cells	No	1/10 HPF	No
